# Case 1/2016 - Aortic Coarctation and Atrial Septal Defect submitted to Percutaneous Repair in Adult Patient

**DOI:** 10.5935/abc.20160008

**Published:** 2016-01

**Authors:** Edmar Atik, Raul Arrieta, Roberto Kalil Filho

**Affiliations:** Hospital Sírio Libanês, São Paulo, SP - Brazil

**Keywords:** Heart Defects, Congenital, Heart Septal Defects, Atrial, Aortic Coarctation, Cardiac Catheterization.

Clinical data: Heart murmur was identified at auscultation in childhood, but the diagnosis of aortic coarctation associated with atrial septal defect was attained when the patient was 15 years old. The patient practiced regular physical activity and did not report symptoms for up to 5 years, when he became sedentary. He received specific anti-hypertensive medication.

Physical examination: patient was eupneic, acyanotic, obese, ample pulses in the upper limbs and decreased in the lower limbs. Weight: 113 kg; height: 177 cm; Body Mass Index (BMI): 36.1 kg/m^2^; RUL BP was the same in the LUL, 149/89 mmHg; right inferior limb BP = 113/77 mmHg; Heart rate (HR): 82 bpm; oxygen saturation of 95%. The aorta was clearly palpable at the suprasternal notch.

The apex beat was not palpable in the precordium and there were no systolic impulses in the left sternal border (LSB). Normal heart sounds; constant split second sound and rough systolic murmur + / ++ / 4, was heard in the upper LSB. The liver was not palpable.

## Complementary Examinations

**Figure 1:**
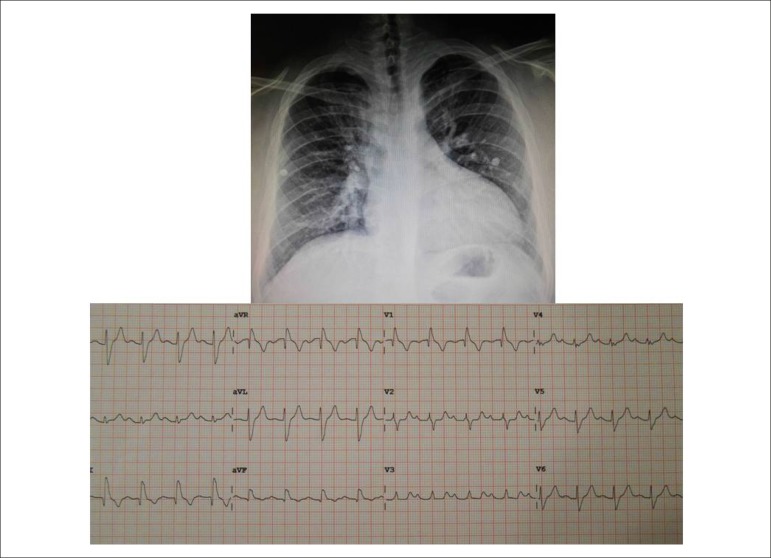
Chest X-ray showing an enlarged cardiac silhouette at the expense of the right ventricular border, with increased pulmonary vasculature, especially in the pulmonary hila. The aorta is not bulging despite the coarctation, but there is hyperrefringency on the edge of the ribs. The electrocardiogram highlights signs of first-degree atrioventricular block and complete right bundle-branch block.

The Electrocardiogram showed sinus rhythm, first-degree atrioventricular block and complete right bundle-branch block. A P: +50º, AQRS: +115º, AT: 0º. QRS Duration: 0.14", PR 0.21 ms (Figure 1).

Chest x-ray showed a slightly enlarged cardiac area (cardiothoracic index of 0.61) at the expense of the right ventricular border. Pulmonary vasculature was increased and the medial border was rectified. There was no aortic dilatation, but hyperrefringency on the lower edges of the ribs was observed (Figure 1).

The echocardiogram showed marked right heart chamber dilation (right ventricle - RV 52 mm), a slight increase of the left atrium (45 mm), systolic blood pressure of 44 mmHg in the RV. The ascending aorta was normal (37 mm) and showed no myocardial hypertrophy (septum/posterior wall of 11 mm). Left ventricular function (75%) and size (39 mm) were normal. TEE showed major discontinuity of the atrial septum at two points, of 8 and 26 mm each, with edges present in the entire contour. No abnormalities were identified in the aorta.

**Figure 2:**
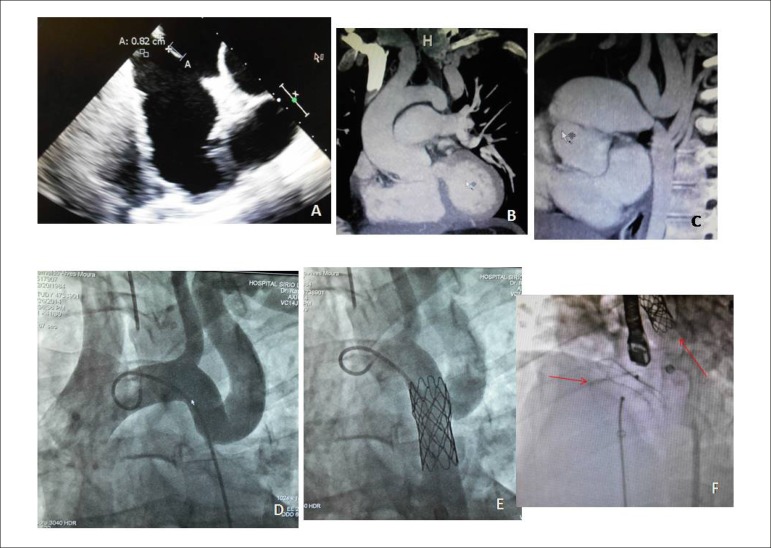
Transesophageal echocardiography shows two ASDs, measuring 8 and 26 mm, in A. Magnetic resonance imaging clearly depicts, in B and C, the aortic coarctation after the left subclavian artery with exuberant collateral circulation into the descending aorta; at the angiography in D, extreme aortic coarctation showing actual aortic disruption after the left subclavian artery and the 15 x 40 mm stent placed at this site, in E; and closure of the ASD using the CERA(™) device in F, together with stent implant in the aorta (arrows).

CT angiography of the aorta, performed after clinical suspicion of aortic coarctation, confirmed the diagnosis, immediately distal to the left subclavian artery, with paravertebral, mediastinal and intercostal artery collaterals, which caused opacification of the descending aorta, with 16 mm in diameter. The internal thoracic arteries and the supra-aortic trunks were enlarged. Marked right chambers and pulmonary trunk enlargement was also observed (Figure 2).

Clinical diagnosis: large atrial septal defect and marked aortic coarctation at the isthmus region, adjacent to the left subclavian artery, without ascending aortic dilatation, with no myocardial hypertrophy and with exuberant collateral circulation.

Clinical reasoning: the clinical elements of aortic coarctation and ASD are easily identifiable, represented by contrast of pulses and blood pressure between limbs in the first abnormality, and in the second, high murmur at the LSB, right bundle-branch block and increased pulmonary vasculature. It is evident that, even in heart disease with long-term pressure overload, there is no myocardial hypertrophy, or signs of electrical overload and with few symptoms. These aspects originate from the evolutionary development of efficient collateral circulation.

Differential Diagnosis: the association of the two defects is unusual, making clinical reasoning difficult, even though the abovementioned elements are indicative of the defects. Aortic obstruction in an adult patient, in general, also result from prior aortitis, as seen in Takayasu's disease, Kawasaki, in connective tissue diseases and infectious diseases such as syphilis.

Conduct: Considering the long-standing systolic effects of arterial hypertension, even without myocardial hypertrophy, surgical indication is mandatory, aimed at relieving arterial obstruction, which leads to the onset of myocardial fibrosis, heart failure, arrhythmias and early death. It was decided to perform dilation of the isthmus region through interventional catheterization from the right femoral artery using a 40 x 15 mm stent, encompassing the left subclavian artery origin. The pressures before the procedure, were 30/12 in the RV; in the PT, 30/15-20; ascending aorta, 140/ 80-100; descending aorta, 90/60-70. There was immediate BP normalization and equalization in the ascending and descending aorta (128/88-101 mmHg) after adequate dilation of the region (Figure 2). The patient reported easier breathing and well-being. The systolic murmur of the relative pulmonary stenosis decreased in intensity after the subsequent ASD closure using a 36 CERA(tm) device.

Comments: aortic coarctation can manifest later in life, in adulthood, in view of the collateral circulation development that supplies the descending aorta, therefore lowering blood pressure in the upper limbs and preventing progression to myocardial hypertrophy. Similarly, the atrial septal defect, being an abnormality with right cavity volume overload, becomes so tolerable that the discovery of the disorder in adulthood becomes even incidental, even after uncomplicated pregnancies.

The association of these defects is unusual and develops independently without significant interference from each other. The percutaneous therapeutic approach has become a reality to the point that, in this patient, it was enthusiastically performed in spite of the blind-end aortic coarctation contiguous to the left subclavian artery. The simultaneity of the percutaneous procedures has been scarcely reported in the literature.^1^ This case exemplifies a successful simultaneous percutaneous procedure.
